# An Unsupervised Learning Approach for Wayside Train Wheel Flat Detection

**DOI:** 10.3390/s23041910

**Published:** 2023-02-08

**Authors:** Mohammadreza Mohammadi, Araliya Mosleh, Cecilia Vale, Diogo Ribeiro, Pedro Montenegro, Andreia Meixedo

**Affiliations:** 1 CONSTRUCT—LESE, Faculty of Engineering, University of Porto, 4200-465 Porto, Portugal; 2 CONSTRUCT, School of Engineering, Polytechnic of Porto, 4249-015 Porto, Portugal

**Keywords:** wheel flat detection, wayside condition monitoring, train-track interaction, unsupervised learning

## Abstract

One of the most common types of wheel damage is flats which can cause high maintenance costs and enhance the probability of failure and damage to the track components. This study aims to compare the performance of four feature extraction methods, namely, auto-regressive (AR), auto-regressive exogenous (ARX), principal component analysis (PCA), and continuous wavelet transform (CWT) capable of automatically distinguishing a defective wheel from a healthy one. The rail acceleration for the passage of freight vehicles is used as a reference measurement to perform this study which comprises four steps: (i) feature extraction from acquired responses using the specific feature extraction methods; (ii) feature normalization based on a latent variable method; (iii) data fusion to enhance the sensitivity to recognize defective wheels; and (iv) damage detection by performing an outlier analysis. The results of this research show that AR and ARX extraction methods are more efficient techniques than CWT and PCA for wheel flat damage detection. Furthermore, in almost every feature, a single sensor on the rail is sufficient to identify a defective wheel. Additionally, AR and ARX methods demonstrated the potential to distinguish a defective wheel on the left and right sides. Lastly, the ARX method demonstrated robustness to detect the wheel flat with accelerometers placed only in the sleepers.

## 1. Introduction

Nowadays, due to the increasing importance of railway transportation infrastructures, many studies have been conducted on their cost-effectiveness, particularly in terms of operation and maintenance costs [1,2,3]. One of the main responsible for the structural degradation of the railway infrastructure, particularly the track, is the operating rolling stock [4,5]. Therefore, an efficient and reliable condition assessment of the rolling stock is crucial for any infrastructure manager.

Many types of damage can affect a train’s operational performance and one of the most important is defective wheels, which include two categories of defects, localized defects in the wheel tread (e.g., wheel flat, spalling and shelling), and defects around the complete wheel perimeter (e.g., wheel corrugation and polygonal wheel).

Wheel flats are the most common type of defect in train wheels and remarkably affects running safety and causes significant damage to the infrastructure, namely the rails and sleepers, due to the higher impact forces induced in the track [6]. The initial cause for the wheel flat is the friction between the wheel and rail due to braking forces, as friction can change the shape of the exterior perimeter of the wheel from round to flat. The wheel flat length is the standard for wheelset maintenance, as stated in the General Contract for the Use of Wagons [7]. For a wheel diameter larger than 840 mm, and in the presence of flat lengths greater than 60 mm, the wheelset should be immediately replaced. Detecting defective wheels at an early stage is recommended to maintain safety, stability, and minimize maintenance costs. 

To do this, an automated approach must be developed that can clearly distinguish between a healthy and damaged wheel. Therefore, finding effective methods for the early detection and identification of wheel flats is of great interest to railway administrations and rolling stock operators.

In the last few decades, researchers have proposed several onboard and wayside systems for detecting wheel defects in operation conditions, most based on the concept that the interaction force between the train and the track increases in a defective wheel [8,9]. Many onboard techniques are based on vibration, acoustic, image detection, and ultrasonic technologies [10,11]. Nevertheless, all wheels must be equipped with sensors for comprehensive diagnosis and effective wheel condition management. The high cost and maintenance problems of this method make it rarely used. Moreover, onboard detection methods are commonly used to monitor track conditions.

Alternatively, wayside measurement systems are currently the preferred solution to identify wheel flats since all wheels are evaluated during the train passage at the specific system location [12,13,14]. Previous research has been focused on advanced signal processing methods to eliminate signal interference and spotlight the faulty signal patterns of wheel flats. Jiang et al. [15] used the empirical mode decomposition (EMD) method to divide the signal into several intrinsic mode functions (IMF) which separates the faulty signal mode from interferences. Amini et al. [13] proposed a method based on time–spectral kurtosis (TSK) to reduce the effect of noise and highlight the faulty signal patterns of wheel flats. Mosleh et al. [16] proposed a method to distinguish a defective wheel from a healthy one based on the envelope spectrum method. Krummenacher et al. [17], by measuring the vertical wheels’ force and using a sensor system permanently installed on the railway track, proposed two machine-learning methods to automatically detect a defective wheel during operation. These methods learn different types of wheel defects and predict whether a wheel has a defect. Yi-Qing et al. [18] developed a probabilistic Bayesian method using trackside strain sensors for the online condition monitoring of the wheels. They found that only using monitoring data from a single sensor may produce false-negative results, but with the data from all the deployed sensors could provide more accurate diagnostic results. 

Typically, the phases for damage identification methods are related to data acquisition, feature extraction, feature normalization, data fusion, and feature classification [1,19]. The process of transforming time series data into alternative information, where the correlation with damage is easily visible, is known as feature extraction [20,21]. Typically, the auto-regressive model (AR) [5], auto-regressive model with exogenous input (ARX) [19], principal component analysis (PCA) [22], and continuous wavelet transform (CWT) [8] are employed to extract the damage-sensitive features using the dynamic responses. 

One of the main challenges to detect a damaged wheel is to remove the environmental and operational effects from the dynamic responses to obtain features that are mainly sensitive to damage but insensitive to environmental and operational changes (EOVs). Therefore, to reduce the variation caused by EOVs and enhance the sensitivity to damage, feature normalization is performed by using various linear and non-linear correction models, such as, PCA [23], kernel principal component analysis (KPCA) [24], non-linear principal component analysis (NLPCA) [25], and factor analysis (FA) [26].

For feature fusion and dimension reduction, several algorithms, including neighborhood-preserving embedding (NPE) [27], neural networks [28], Mahalanobis distance [29], manifold-learning methods [30], and kernel-based methods [31], have recently been employed. The capability of the Mahalanobis distance to capture the variability in multivariate datasets has led to the widespread use of this technique [23]. This method has been used in multiple research studies with excellent results as it increases the sensitivity to the damage and can integrate data from various sensors [32].

In recent years, machine-learning (ML) approaches in combination with advanced signal processing methods have been applied for feature classification to differentiate a healthy wheel from a defective one [18,33]. Unsupervised and supervised learning are two different types of ML techniques. Unsupervised learning involves finding hidden structures in unlabeled data to classify them into meaningful categories. On the other hand, supervised learning assumes that a database’s categories or hierarchy of the database are known in advance. Researchers have recently investigated supervised and unsupervised approaches for classifying data based on dedicated features, including unsupervised methods, such as, k-mean [1], self-organizing maps (SOM) [34], and cluster analysis, as well as supervised methods, such as, naive Bayes classifiers [35] and k-nearest neighbor (kNN) classifiers [36].

Most of the previous research on wheel flat detection is based on engineering field tests. However, numerical analysis is very useful for understanding the mechanism and physical consequences based on dedicated models. Additionally, models can be used for deeper comprehension and prediction in situations that cannot be reproduced in experimental tests. For example, external elements, such as noise, environmental disturbances, measurement errors, and electromagnetic interferences, easily influence the measurement process and may affect the results, causing a decrease in measurement accuracy. Additionally, numerical simulation makes it possible to define each unknown variable separately to check how it affects the results.

It should be highlighted that the initial research on this topic was developed by Mosleh et al. [5,8], who proposed an automatic wheel flat identification method based on shear and accelerometer time series evaluated on the rails. It should be noted that the CWT [8] and AR [5] methods have been used separately in each research to extract features. However, none of these studies compared the accuracy of different features. Therefore, one of the novelties of this research is the comparison of the accuracy of four different feature extraction techniques using an unsupervised learning methodology to automatically detect a defective wheel, which is a clear step forward in terms of the effectiveness of the proposed method and allows full implementation for real-world application. Therefore, a 3D numerical dynamic model of a vehicle–track coupling system was used for this purpose. The features were extracted by applying the AR, ARX, PCA, and CWT models to the measurement records. Moreover, PCA, as well as Mahalanobis distance, were used for feature modeling and data fusion, respectively. Finally, outlier and cluster analyses were applied for feature classification. The following significant contributions can be highlighted from this research work:

(1) Development of an unsupervised data-driven methodology using acceleration responses on the rail for detecting defective wheels from healthy ones;
(2) Implementation of AR, ARX, CWT, and PCA for feature extraction from multiple sensors to transform the time series measurements into damage-sensitive features, where the correlation with the damage can be more easily observed;
(3) Analysis of the performance of the four feature extraction methods considering the different number and locations of the sensors on the rails;
(4) Comparison of the sensitivity of the proposed methodologies to the side (left vs. right) of the defective wheel in a train axle;
(5) Evaluation of the effectiveness of the proposed method with respect to the minimalist layout of sensors;
(6) Improvement in wheel flat detection by applying a two-stage fusion process: in the first step, the features from each sensor are merged and, in the second stage, the multi-sensor information is fused to enhance the sensibility to the damage.

## 2. Numerical Simulation

### 2.1. Train–Track Dynamic Interaction

**Figure 1:**
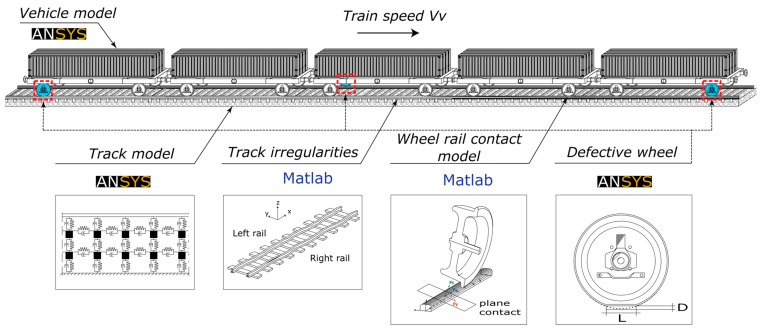
Numerical modeling of the train–track system.

In this study, by using in-house software vehicle–structure interaction (VSI), the simulations for numerical train–track dynamic interaction were carried out. The vehicle–structure interaction analysis is explained and validated in detail in the work of Montenegro et al. [37] and has been used in several applications [5,16]. A 3D wheel–rail contact model couples the train to the track using Hertzian theory [38], to compute normal contact forces, and USETAB routine [39], to compute the tangential forces caused by rolling friction creep. The structural matrices from the structure (in this case, the track) and the vehicle, previously modeled in a finite element program (FE), were imported into this numerical tool, which was developed in MATLAB [40]. Although these subsystem models were initially created individually, the VSI program connects them using a fully linked technique [37]. Figure 1 represents the graphical representation of this procedure.

The software ANSYS [41] was used to simulate the track. Beam elements were used to model the rails and sleepers, while spring–dashpot components were used to simulate the behavior of the flexible layers, i.e., the ballast, fasteners/pad, and mass point components to account for the ballast’s mass as shown in Figure 1. The train was composed of five wagons of Laagrss type, each one with two axles, had also been modeled in ANSYS [41] through a multibody formulation, using mass point elements located at the center of gravity of each body, specifically the car body, and wheelsets, to simulate their mass and inertial effects. Rigid beams were also used to consider the rigid body movements of the vehicle. The characteristics of both the track and train models are fully described in the work of Mosleh et al. [16,42].

### 2.2. Virtual Wayside System

**Figure 2:**
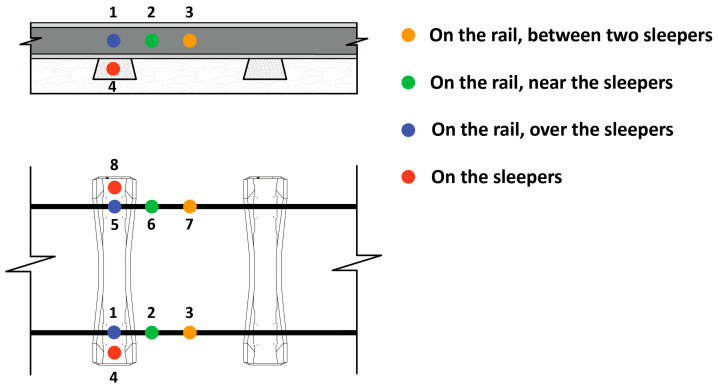
Virtual wayside monitoring system.

A set of eight accelerometers were considered along the track as part of the wheel flat-detecting system. Figure 2 depicts the position of the sensors in the proposed virtual wayside monitoring system. Measurement points 1 to 4 simulate the position of the accelerometers located on the right side of the track, particularly on the rail and on the sleepers; otherwise, measurement points 5 to 8 represent the sensors located on the left side of the track. In Section 4, accelerometers 1–4 were selected to depict the results. One of the main advantages of the proposed method compared to previous approaches [16,42,43] is that there is no need to install a series of sensors on the rail to monitor the whole perimeter of the wheel. Only a minimalist set of sensors are sufficient to detect a defective wheel. 

### 2.3. Baseline and Damaged Scenarios

For testing and validating the automatic wheel flat-identification method proposed in this work, baseline (undamaged) and damaged wheel scenarios were considered. After validation, this method can reproduce real experimental data, from different types of trains with various wheel defects, running at different speeds on the rail track with distinct rail irregularities profiles.

As shown in Figure 1, for damaged scenarios, three defective cases are considered, particularly ones located on: (i) the right wheel on the front wheelset of the first wagon (Damage 1), (ii) the left wheel of the rear wheelset of the third wagon (Damage 2); (iii) right wheel of the rear wheelset of the fifth (last) wagon (Damage 3). The lower and upper bounds of the flat length are defined by uniform distributions U (50, 100) for the three defective wheels. The wheel flat depth (D) is defined by the following expression [41]:

$$D=\frac{L^{2}}{16R_{w}}$$

where L is the flat length and R_w_ the radius of the wheel.

The vertical profile deviation of the wheel flat (Z) is defined as follows [41]:

$$Z\;=-\frac{D}{2}\;\left(1-\cos\frac{2\pi x}{L}\right).H\left(x-\left(2{\pi R}_{w\;}-L\right)\right),\;\;\;0\leq x\leq \;2{\pi R}_{w\;}$$

where H represents the Heaviside periodic function, and x is the coordinate aligned with the track longitudinal direction.

Wheel-rail contact force values are significantly affected by imperfections in a real-condition environment, where the rails are not completely smooth. Although these irregularities are very small, they should be considered in the numerical analyses. Four real unevenness track profiles are taken into consideration in this study. The selected unevenness profiles of the rail are measured on the Northern Line of the Portuguese Railway network based on the track inspection vehicle EM120 and according to the details provided by Mosleh et al. [14]. The total length of the simulation was 1000 m.

To evaluate the proposed methodology, the accelerations on eight positions of the rail were evaluated in both baseline (undamaged) and damaged scenarios. The baseline condition corresponds to a train passing with healthy wheels, while the damaged scenarios correspond to the passage of trains with defective wheels. Table 1 summarizes the assumptions for damaged and baseline scenarios, as well as the number of numerical analyses performed for each scenario.

**Figure 3:**
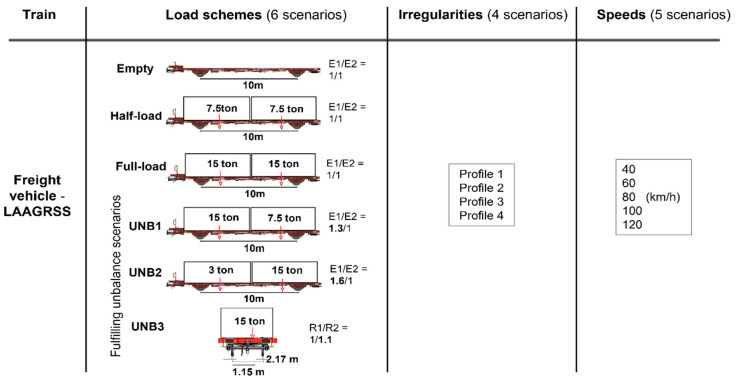
Baseline scenarios.

Figure 3 presents the baseline scenario for which 113 simulations were performed considering a freight train comprising five wagons. Six different types of loading schemes were considered: (i) full-loaded train; (ii) half-loaded train, (iii) empty train, as well as trains with unbalanced loads in the transversal and longitudinal directions, namely (iv) UNB1, (v) UNB2 and (vi) UNB3. According to UIC loading guidelines [44] different unbalanced loading schemes were defined for the wagon model, where the cargo gravity center was offset in longitudinal and transversal directions.

**Figure 4:**
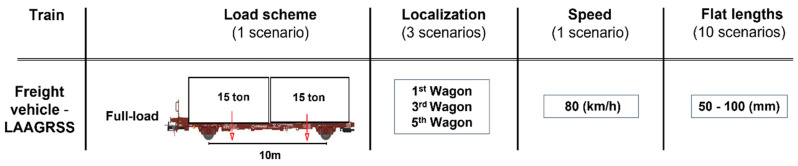
Damaged scenarios.

Figure 4 illustrates the damaged scenarios for 30 simulations which were implemented considering several combinations of flat properties for defective wheels. As mentioned before three defective cases were considered in this study, namely Damage 1, Damage 2 and Damage 3, which are located on the 1st, 3rd and 5th wagons, respectively. In total, 10 analyses were performed for each damaged wheel (Damages 1, 2 and 3) and the speed was considered equal to 80 km/h. Moreover, a sampling frequency of 10 kHz was used to evaluate acceleration signals for both baseline and damage scenarios.

The numerical signal was then polluted with artificial noise (5% of the amplitude) based on the maximum response of the signal for a more realistic reproduction of the measured rail response. 

**Figure 5:**
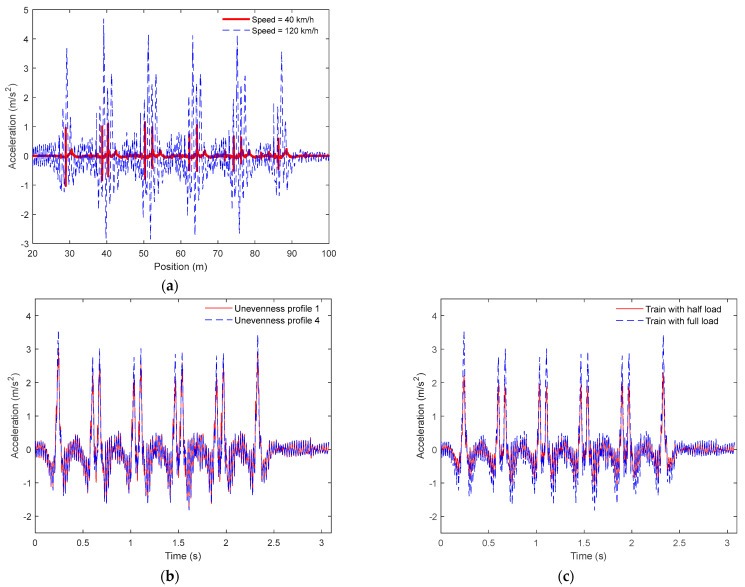
Acceleration time series for sensor 3 for a freight train considering a healthy wheel (baseline scenario): (**a**) influence of vehicle speed, (**b**) influence of the unevenness profile, (**c**) influence of the loading schemes.

In Figure 5 are shown examples of acceleration time series for baseline scenarios obtained in sensor 3, located on the rail. These figures show the influence of different loading schemes, train speeds, and unevenness profiles on the track response. All-time series were filtered using a low-pass Chebyshev type II digital filter with a cut-off frequency of 500 Hz.

Figure 5a demonstrates the relevant influence of the train speed on the evaluated acceleration, and the need to consider various train speeds for identifying wheel flats. Additionally, as shown in Figure 5b, both unevenness rail profiles induced similar acceleration responses. Finally, Figure 5c shows that both loading schemes affect the track responses particularly on the peak acceleration values.

## 3. Unsupervised Learning Methodology for Wheel Flat Detection

**Figure 6:**
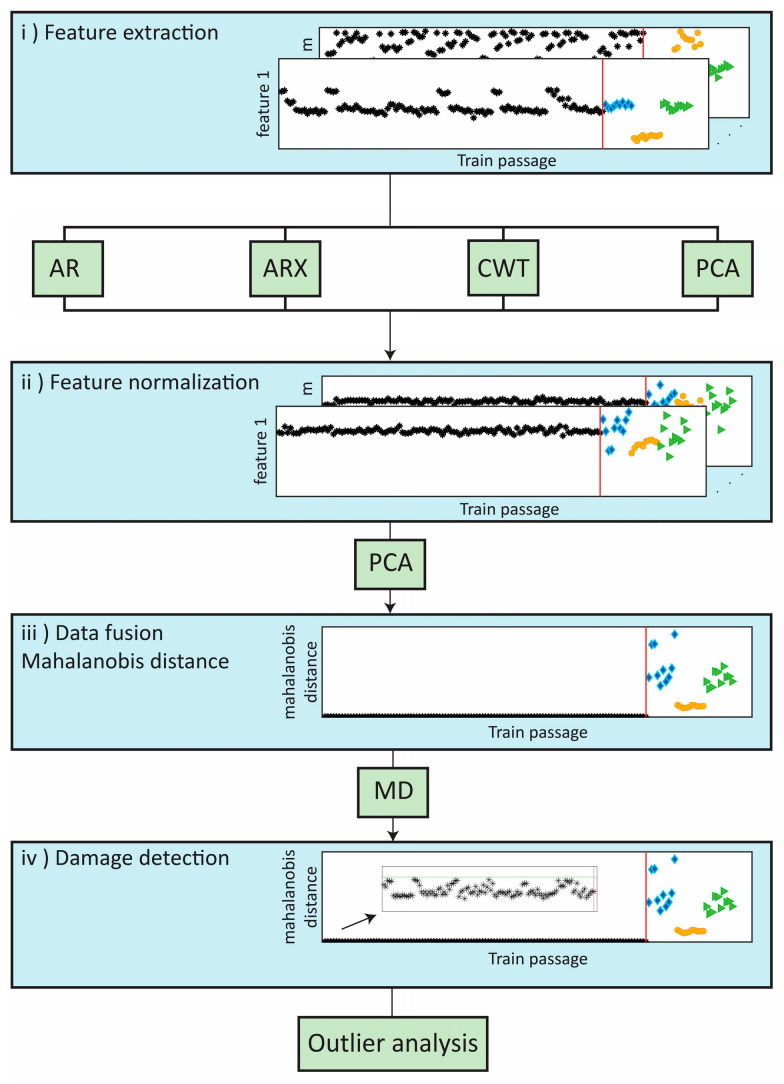
Methodology of the automatic detection of wheel flats.

The purposed methodology for the automatic detection of wheel flats presented in Figure 6 includes four steps, particularly:

1. Features extraction: application of four advanced data-driven models, including the continuous wavelet transform (CWT), auto-regressive model (AR), principal component analysis (PCA), and ARX to extract the damage-sensitive features from the time series;
2. Feature normalization: normalization of the extracted features by the principal component analysis (PCA) method to increase the sensitivity to damage and remove environmental and operational variations (EOVs);
3. Data fusion: implementation of a Mahalanobis distance (MD) to merge the features derived from each sensor and detect wheel defects more effectively. In the first stage, the features from each sensor are merged and, in the second stage, the multi-sensor information is fused to enhance the sensibility to the damage [26,32];
4. Outlier analyses: upon completion of the previous step, a damage indicator (DI) is generated for each train passage; to distinguish each DI into a defective or a healthy wheel a statistical-based approach is used, in particular, an inverse cumulative distribution function that allows estimating a statistical confidence boundary (CB).

The theoretical framework of each technique implemented within the methodology is available in the authors’ previous publications [1,5,8,19,45].

## 4. Application of the Methodology of Wheel Flat Detection to a Freight Train

This section presents the application of the unsupervised learning methodology of wheel flat detection to the case of a freight train and considers different feature extraction methods, namely the AR, ARX, CWT and PCA. The purpose of this comparison was to assess the sensitivity to damage of each extraction method.

### 4.1. Feature Extraction

Damage-sensitive feature extraction from dynamic signals is the first step of the automatic damage detection methodology. The main goal of this step is to reduce the dimensions of the three-dimensional dynamic features matrices 143−by−q−by−n, in which, 143 is the total number of scenarios, including 113 baseline scenarios and 30 damage scenarios, q is the number of sensors (four sensors) and n is the dimension of dynamic time-histories (70,000). For this purpose, the extraction of features sensitive to the effects of wheel flats was performed by considering auto-regressive model (AR), principal component analysis (PCA), continuous wavelet transform (CWT), and auto-regressive model with exogenous input (ARX).

#### 4.1.1. AR Model

**Figure 7:**
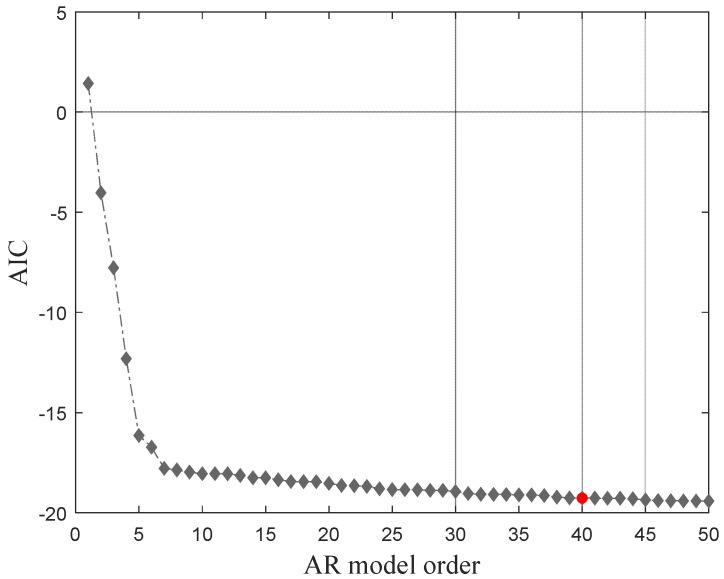
AR model order definition.

Several AR models were analyzed to determine the appropriate model order based on the Akaike information criteria (AIC), particularly the orders between 1 and 50. The AIC function for 30 damaged scenarios is shown in Figure 7. It can be concluded that, as the model’s order increases, the AIC values tend to stabilize, which demonstrates that after a model order of 40, higher orders do not yield relevant information.

**Figure 8:**
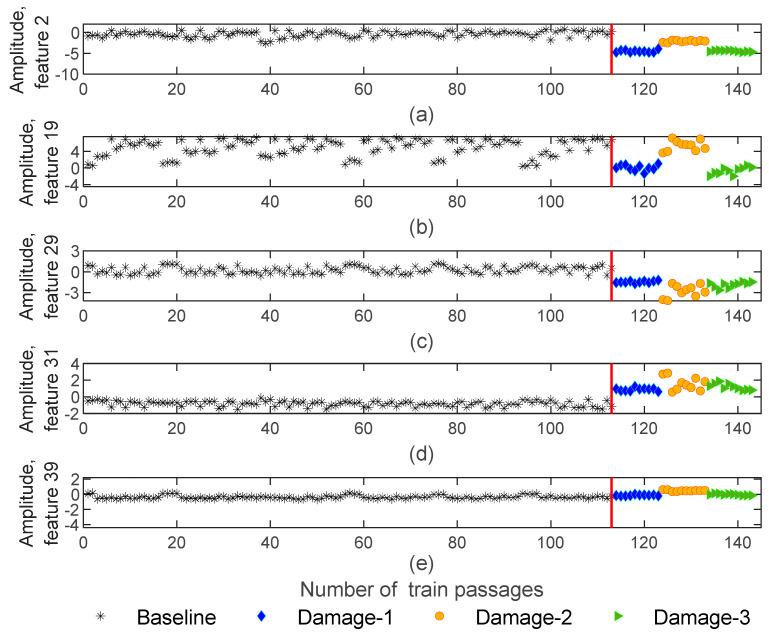
AR—feature extraction for all 143 baseline and damaged scenarios for accelerometer 3: (**a**) amplitude for feature 2, (**b**) amplitude for feature 19, (**c**) amplitude for feature 29, (**d**) amplitude for feature 31, (**e**) amplitude for feature 39.

Extracted features from dynamic responses by implementing the AR method are obtained in 143−by−4−by−40 matrices which means that the number of features is significantly reduced from 70,000 to 40. Figure 8 illustrates five of the features obtained using the AR method for sensor 3. As shown in this figure, a particular sensitivity pattern is recognized for damaged scenarios, in such a way that, amplitude is sensitive to the side of the defect (right or left wheels). As an example, Figure 8b shows the amplitude of feature 19 for the 1st and 5th wagons with blue and green colors. Note that the defect is located on the right-side wheels for the 1st and 5th wagons, while for the 3rd wagon, the defect is placed on the left-side wheel which is presented in an orange color. It is noticeable that the amplitude is sensitive to the side of damage (left or right wheel). Additionally, in Figure 8d, due to the comparison of the amplitude variations between damage and baseline scenarios, it is possible to state a significant difference between healthy and damaged wheels. For other features, this difference is not so significant or visible, as is the case in Figure 8e.

#### 4.1.2. ARX Model

**Figure 9:**
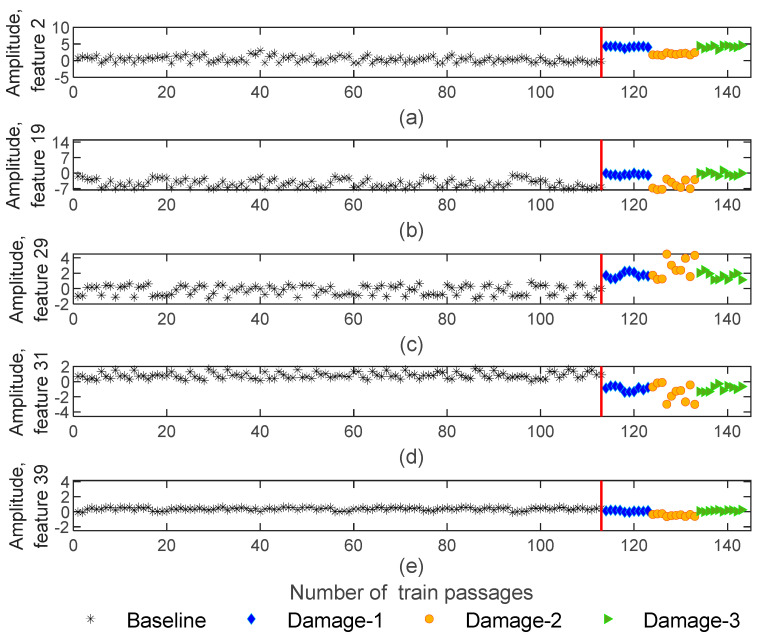
ARX—feature extraction for all 143 baseline and damage scenarios for accelerometer 3: (**a**) amplitude for feature 2, (**b**) amplitude for feature 19, (**c**) amplitude for feature 29, (**d**) amplitude for feature 31, (**e**) amplitude for feature 39.

The auto-regressive model with exogenous input (ARX) is the second technique that was used to extract dynamic damage-sensitive features. This method of time-series analysis can perform a significant fusion while accurately generalizing the information contained in the data by adjusting the ARX (143−by−4−by−80) models. By using the ARX model the number of features is enlarged to 80 in comparison to the AR model. Figure 9 presents five of the features obtained by using the ARX method for sensor 3. As in the AR model, the damage scenarios features are also sensitive to the side of the wheel damage. As an example, in Figure 9b, the blue and green colors corresponding to a defective wheel on the right side of the 1st and 5th wagons, have similar amplitude values and are distinct from the ones associated with the defective wheel on the left side of the 3rd wagon, represented by the orange color. Moreover, as shown in Figure 9d, the difference between the amplitudes between healthy and defective wheels is evident.

#### 4.1.3. CWT

**Figure 10:**
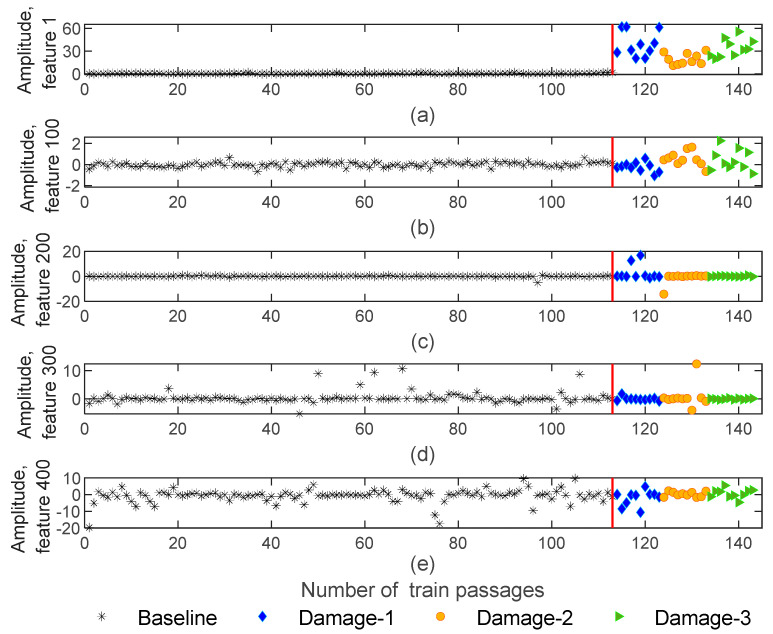
CWT—feature extraction for all 143 baseline and damage scenarios for accelerometer 3: (**a**) amplitude for feature 1, (**b**) amplitude for feature 100, (**c**) amplitude for feature 200, (**d**) amplitude for feature 300, (**e**) amplitude for feature 400.

Another methodology that was implemented for feature extraction to reduce the size of the feature matrices was the continuous wavelet transform. By using CWT, the number of features is decreased from 70,000 to 468 and the obtained features matrices are of size 143−by−4−by−468. Figure 10 represents the extracted features for the CWT method, which shows sensitivity to the damage but not as much as the AR and ARX models. As an example, Figure 10a,b provides evidence that the features are sensitive to damage since their amplitude variation for damage scenarios is higher than for the healthy scenarios. However, for the features shown in Figure 10c,e, the amplitude variation is similar for healthy and defective wheels and the features are not sensitive to the damage. Furthermore, all the extracted features using CWT extraction are not sensitive to the side of the wheel defect.

#### 4.1.4. PCA

**Figure 11:**
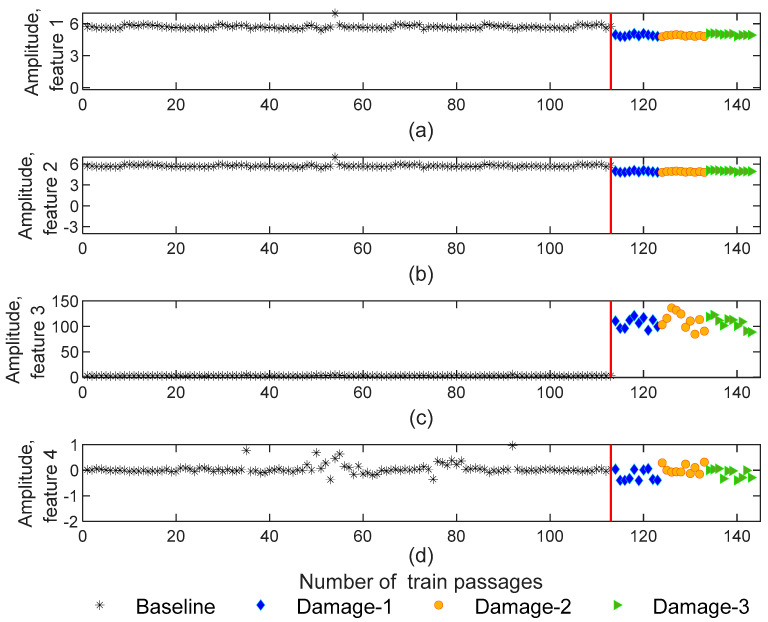
PCA—feature extraction for all 143 baseline and damage scenarios for accelerometer 3: (**a**) amplitude for feature 1, (**b**) amplitude for feature 2, (**c**) amplitude for feature 3, (**d**) amplitude for feature 4.

Data science frequently uses principal component analysis (PCA) to extract features based on the data projection into a new dimensionless subspace. PCA identifies the covariance matrix eigenvectors with the highest values [1,5,8,19]. In other words, the PCA method minimizes the number of features that effectively can capture the most significant of the original features. Thus, the number of extracted features is reduced to four and the matrices of damaged features are generated with 143−by−4−by−4 dimensions. The extracted features using PCA are represented in Figure 11. As shown, for features one and two, the dispersion of amplitude for healthy and defective wheels is almost imperceptible (Figure 11a,b). In turn, as Figure 11c shows for feature three, the amplitude variation of damaged scenarios is higher than baseline scenarios, and the amplitude difference between the damaged and healthy wheels is quite visible. This proves that only specific features have the potential to identify the damage.

### 4.2. Feature Normalization

Data normalization allows to distinguish changes in the features acquired from sensor readings influenced by environmental and operational variations. One of the significant issues in damage detection is the difficulty of isolating environmental and operational disturbances from the observed dynamic properties to obtain features that are primarily sensitive to damage. Without the requirement to measure these actions, implementing a latent variable approach, such as PCA, to the retrieved features may effectively limit the effects of EOVs. In the feature normalization procedure, during the modeling phase, a cumulative percentage of the variance of components with a variance greater than 80% is removed [8].

#### 4.2.1. AR Model

**Figure 12:**
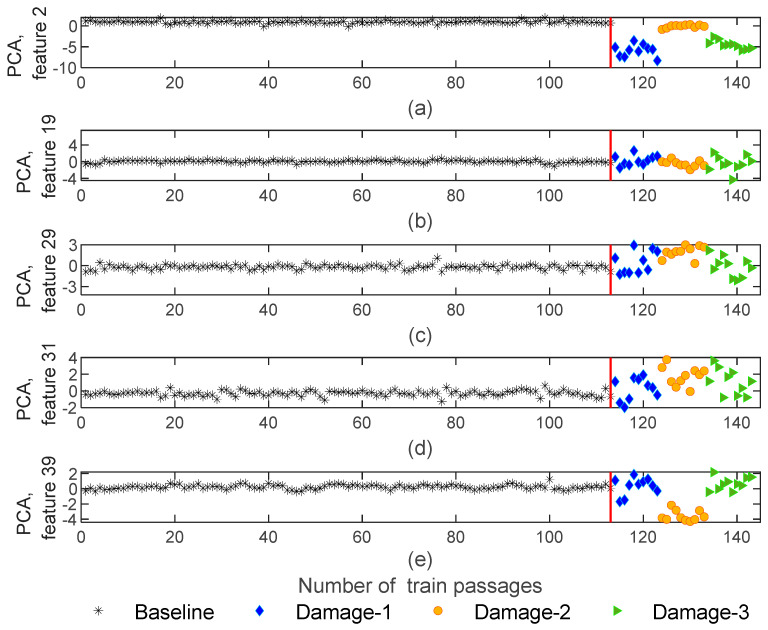
AR—feature normalization for all 143 baseline and damage scenarios for accelerometer 3: (**a**) PCA for feature 2, (**b**) PCA for feature 19, (**c**) PCA for feature 29, (**d**) PCA for feature 31, (**e**) PCA for feature 39.

By implementing the PCA method to AR parameters to normalize the features, for each train passage, a 4−by−40 matrix with PCA-based features was generated. Figure 12 represents 5 features out of 40 for all the 143 baseline and damage scenarios using the AR model. As shown in Figure 12a,d, after removing EOVs, features remain sensitive to damage and significant variations in amplitude can occur between the baseline and damage scenarios. Additionally, it is noteworthy that, after normalization, the extracted features by using the AR model remain sensitive to the side where the wheel defect occurs. As an example, in Figure 12a,e, the variation in amplitude for the 1st and 5th wagons (blue and green colors) are differentiable from the 3rd wagon (orange color). 

#### 4.2.2. ARX Model

**Figure 13:**
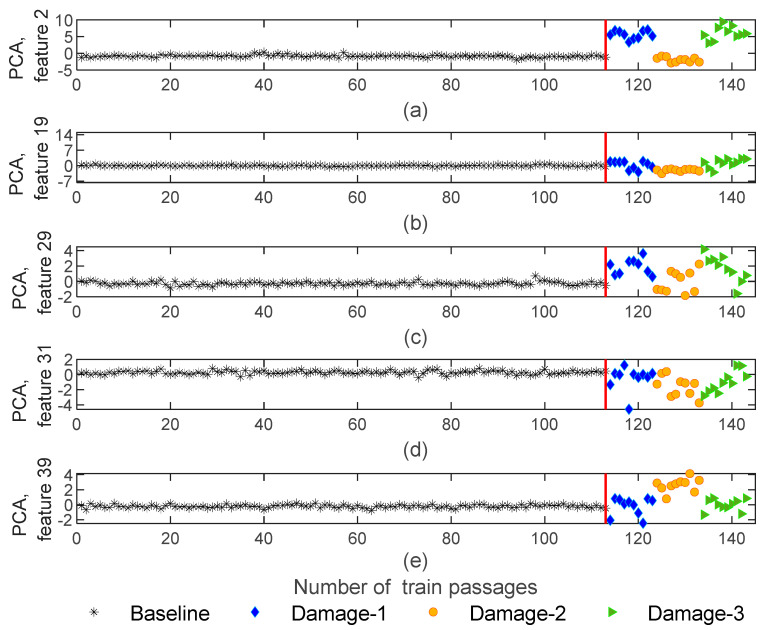
ARX—feature normalization for all 143 baseline and damage scenarios for accelerometer 3: (**a**) PCA for feature 2, (**b**) PCA for feature 19, (**c**) PCA for feature 29, (**d**) PCA for feature 31, (**e**) PCA for feature 39.

Feature normalization was also applied to the features extracted from the ARX method, and as a result, a matrix with dimension 4−by−80 was obtained individually for each train passage. Figure 13 shows that after implementing normalization the ARX features show specific sensitivity to damage. As seen in the examples shown in Figure 13c,e, the wheel defects have a noticeable effect on the variation in the features’ amplitude. Moreover, as with the AR feature, the sensitivity to the side of the damage (left or right defective wheel) is still recognizable in some features after the elimination of the environmental effects, as stated in Figure 13a,e.

#### 4.2.3. CWT

**Figure 14:**
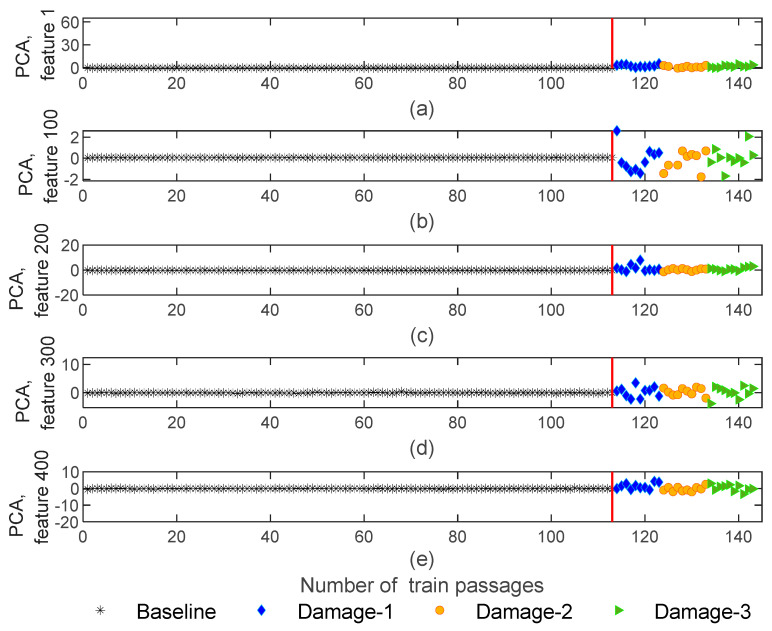
CWT—feature normalization for all 143 baseline and damage scenarios for accelerometer 3: (**a**) PCA for feature 1, (**b**) PCA for feature 100, (**c**) PCA for feature 200, (**d**) PCA for feature 300, (**e**) PCA for feature 400.

Figure 14 shows five of the normalized features which are obtained by using CWT. In contrast to the AR and ARX feature normalization, the CWT normalization has an adverse effect on the sensitivity of the features to damage, and therefore, after normalization, the features are not sensitive enough to wheel defects. As an example, Figure 14a,c,e shows that the PCA-based normalized features lose sensitivity to the defects. Therefore, the different damages cause negligible variations in the amplitude of the feature, and no clear distinction is achieved in relation to the baseline. Additionally, the sensitivity in relation to the side of the damage is not recognizable for the CWT normalized features.

#### 4.2.4. PCA

**Figure 15:**
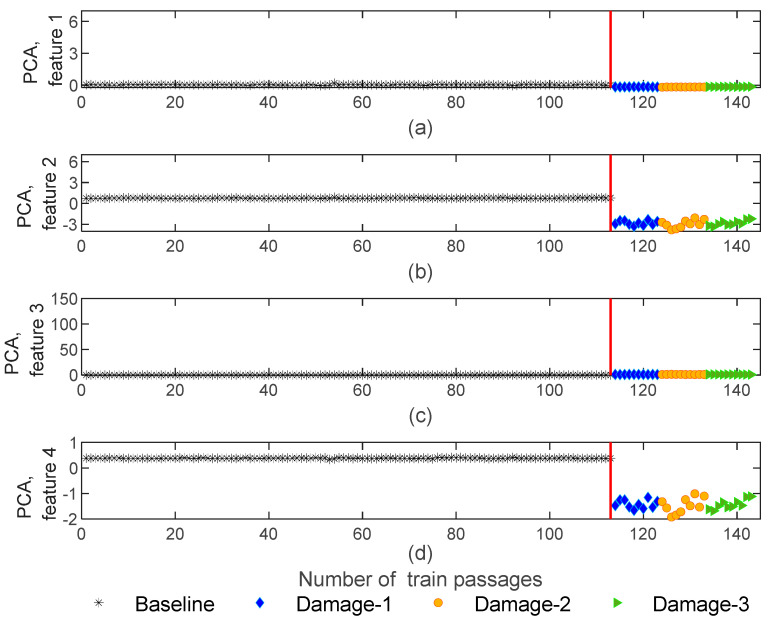
PCA—feature normalization for all 143 baseline and damage scenarios for accelerometer 3: (**a**) PCA for feature 1, (**b**) PCA for feature 2, (**c**) PCA for feature 3, (**d**) PCA for feature 4.

Figure 15 shows that the PCA normalized features are influenced by environmental and operational effects, as shown in the compression of the amplitude’s variation in comparison to the situation before normalization (Figure 11). Moreover, as shown in Figure 15b,d, the variation in amplitude for the damaged scenarios is quite distinguishable from the baseline scenarios, and features after normalization are sensitive to the defects. On the other hand, the PCA normalized features are not sensitive to the side of the wheel defect.

### 4.3. Data Fusion

The results of Section 4.2 show that after the elimination of the environmental and operational effects, the difference between the baseline and damaged scenarios is not sufficient to distinguish healthy from damaged wheels. Therefore, the data fusion process was performed to increase the sensitivity of the features to the defect, and, as a result, a damage index (DI) was achieved for each simulation. Mahalanobis distance (MD) was used to reduce multivariate data into one single DI. To determine the similarities between the damaged and baseline features, the Mahalanobis distance (MD) calculates the distance between defective and healthy wheels, in which shorter distances represent higher similarities. In this step, the MD was obtained for each measurement point and train passage, and therefore, can transform all features into one single damage-sensitive feature. Thus, as a result, a distances vector with dimension 143−by−1 was calculated for every four sensors associated with each feature extraction method.

#### 4.3.1. AR Model

**Figure 16:**
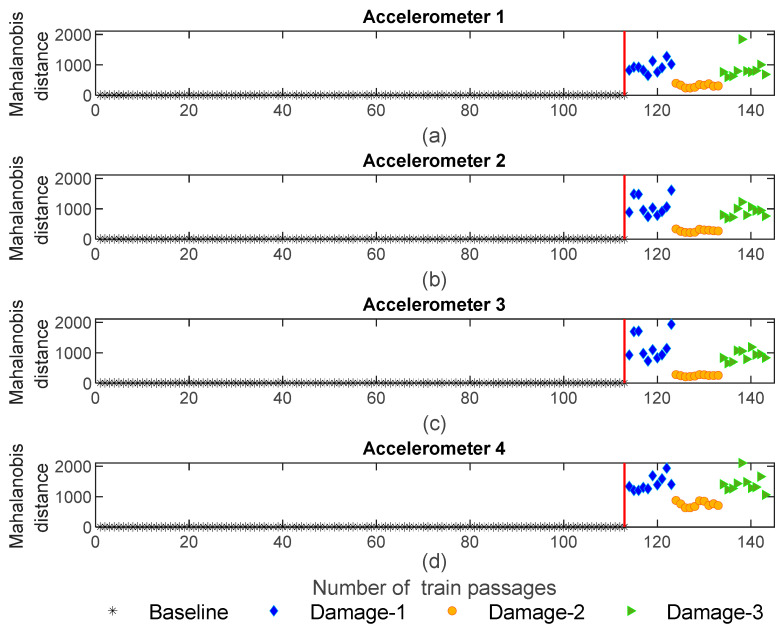
AR—data fusion for all 143 baseline and damage scenarios: (**a**) MD for accelerometer 1, (**b**) MD for accelerometer 2, (**c**) MD for accelerometer 3, (**d**) MD for accelerometer 4.

Figure 16 shows the values for the Mahalanobis distance for accelerometers 1–4 (see Figure 2). It is noticeable that the MD is sensitive to the defects and the variation in MD for defective wheels is higher than for healthy ones. Additionally, the MD is clearly sensitive to the side of the damage, as stated by the train passages of the defective wheel in the 3rd wagon (orange color) which have less amplitude compared with defective wheels on the right side of the 1st and 5th wagons (blue and green colors, respectively). Thus, as illustrated in this figure, it is possible to distinguish the damaged scenarios based on the side of the wheel defect.

#### 4.3.2. ARX Model

**Figure 17:**
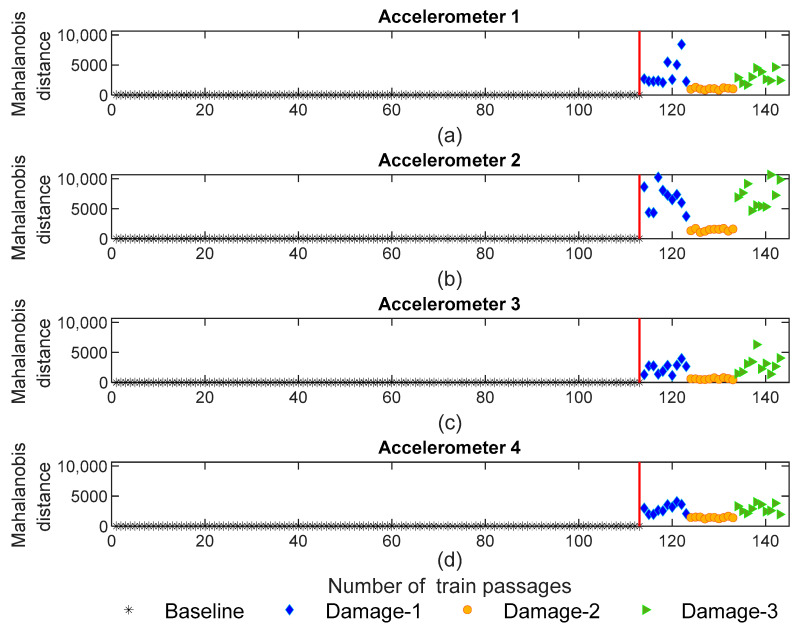
ARX—data fusion for all 143 baseline and damage scenarios: (**a**) MD for accelerometer 1, (**b**) MD for accelerometer 2, (**c**) MD for accelerometer 3, (**d**) MD for accelerometer 4.

The MD values for the ARX-normalized features are presented in Figure 17. It can be observed that the fusion of the features significantly increases the sensitivity to damage, and after the fusion the influence of damages is recognized, as stated by the amplitude of the variation for the damage scenarios which reaches a magnitude of 10,000. From this point, it can also be concluded that defective wheels can be distinguished from healthy ones. Furthermore, as shown in Figure 17, the MD is sensitive to the side of the damage and the defective wheels on the right side (blue and green colors) can be distinguished from the ones on the left side (orange color). Additionally, based on the amplitude values, it is possible to conclude that the features extracted by the ARX model are more sensitive than the ones derived from the AR model.

#### 4.3.3. CWT

**Figure 18:**
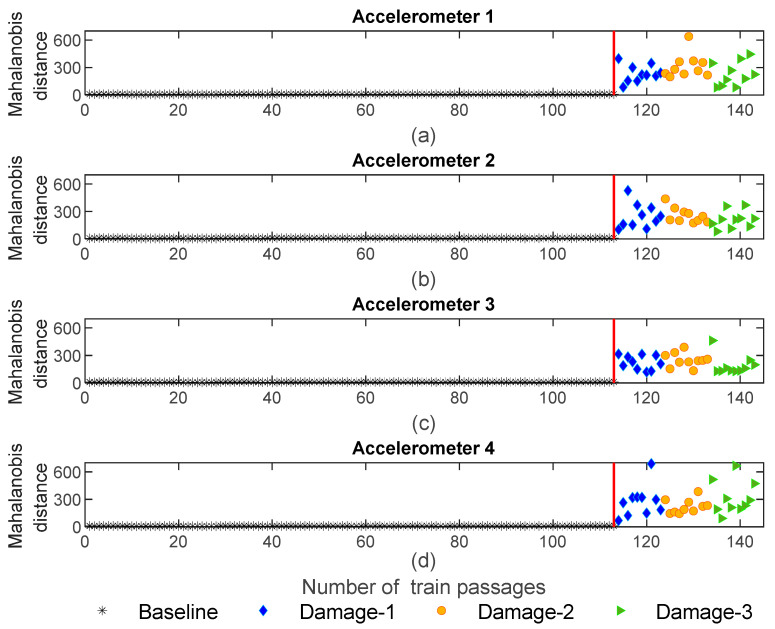
CWT—data fusion for all 143 baseline and damage scenarios: (**a**) MD for accelerometer 1, (**b**) MD for accelerometer 2, (**c**) MD for accelerometer 3, (**d**) MD for accelerometer 4.

Figure 18 shows the MD for sensors 1–4 using CWT feature fusion. As presented in this figure, the amplitude of variation for the damaged scenarios is higher than for baseline scenarios; however, the maximum amplitude of the MD is 600 which is less than the value obtained for AR and ARX. On the other hand, it is noteworthy that, the amplitude of the MD for the defective wheel on the right side has the same range as the damaged wheel on the left side, which means that the MD based on the CWT features is not sensitive to the side of the wheel defect.

#### 4.3.4. PCA

**Figure 19:**
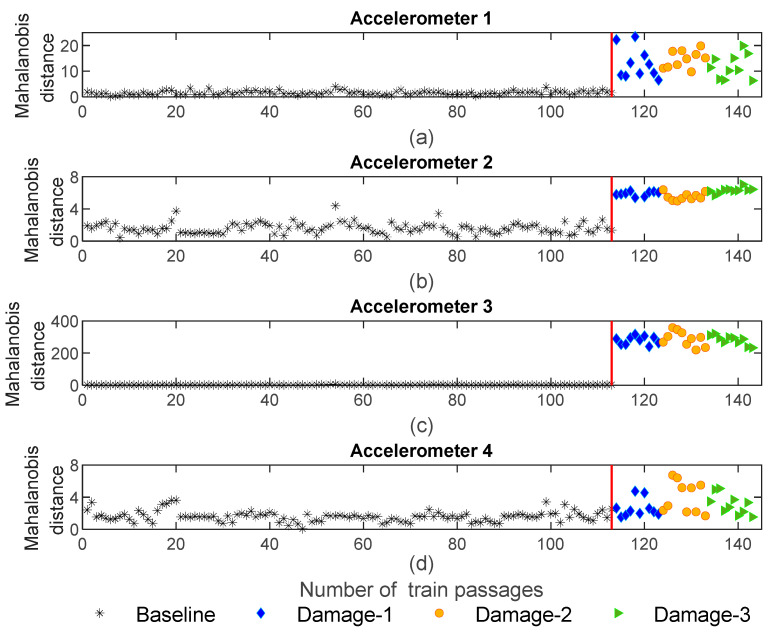
PCA—data fusion for all 143 baseline and damage scenarios: (**a**) MD for accelerometer 1, (**b**) MD for accelerometer 2, (**c**) MD for accelerometer 3, (**d**) MD for accelerometer 4.

Figure 19 shows the Mahalanobis distance based on the PCA extraction method. As shown in Figure 19c, the sensitivity for the MD based on the PCA is less than the AR and ARX models. Additionally, it should be mentioned that like CWT and in opposition to AR and ARX, the MD is not sensitive to the side of the wheel defect since the variation in the amplitude for the MD does not change between the three distinct damage scenarios.

### 4.4. Outlier Analysis

Outlier analysis allows the assessment of how effectively the suggested methodologies distinguish healthy wheels from defective ones for all feature extraction methods without human intervention. In general, the literature presupposes that a chi-squared distribution in n−dimensional space can approximate the Mahalanobis-squared distance. Therefore, a Gaussian distribution can roughly represent the Mahalanobis distance, and an outlier analysis based on a statistical threshold can be performed. The threshold’s significance level is established as equal to 1% [46]. According to this theory, a confidence boundary (CB) for identifying a damage index consisting of an outlier is calculated using the Gaussian inverse cumulative distribution function (ICDF), considering the mean value, $\overset{-}{\mu }$, and standard deviation, σ, of the baseline feature vector. Finally, feature damage indicators equal or greater than the CB are considered outliers (the null hypothesis is rejected).

#### 4.4.1. AR Model

**Figure 20:**
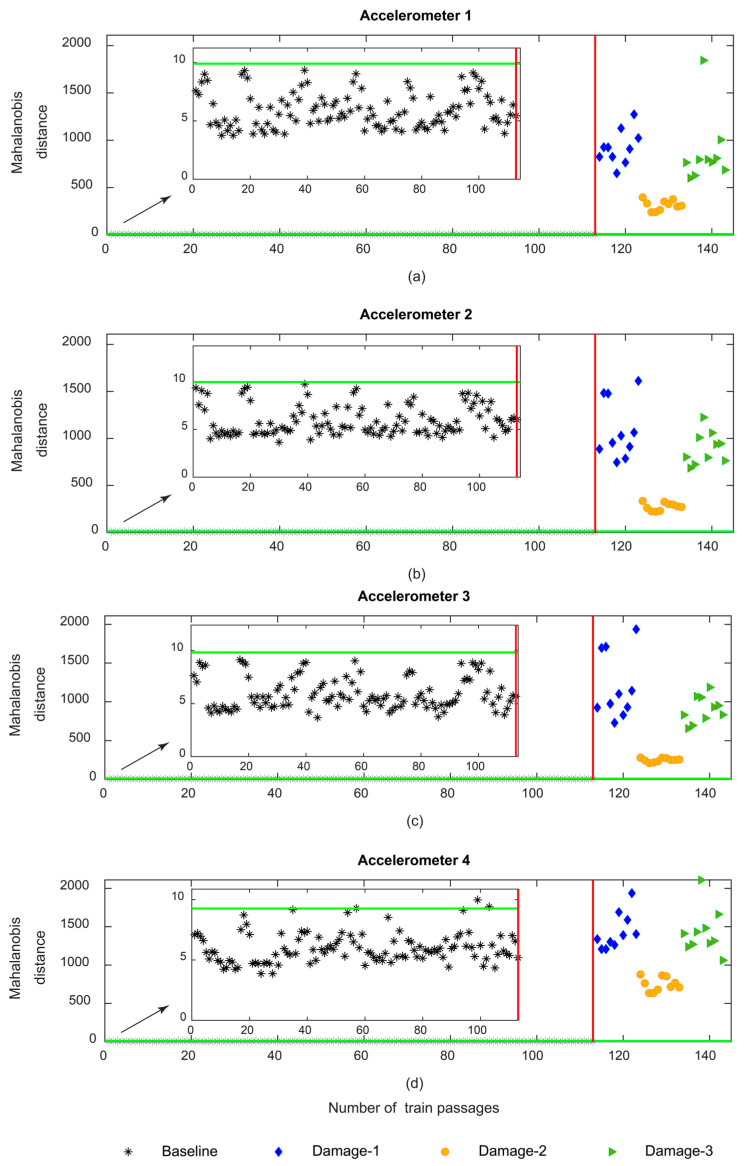
AR—automatic wheel flat damage detection considering the responses from accelerometers 1–4: (**a**) MD for accelerometer 1, (**b**) MD for accelerometer 2, (**c**) MD for accelerometer 3, (**d**) MD for accelerometer 4.

Figure 20 depicts the results of the automatic damage detection system that considers the responses from accelerometers 1–4 using the AR model. This figure indicates that damage detection can be effectively performed using only sensors installed on the rail (between or above sleepers). As an example, according to Figure 20a–c, the damaged wheels are efficiently detected without the occurrence of false-positive cases, and so the healthy wheels can be robustly separated from damage scenarios. Moreover, the distance between the damaged wheels and the CB is sufficiently high; however, for the baseline scenarios this distance is sometimes very close to the CB. On the other hand, in the case of sensor 4 (Figure 20d), located on the sleeper, the damage detection implies some false-positive cases, which means that damage detection is not accurate enough based on the data exclusively derived from accelerometers on the sleeper. Furthermore, by using the AR-derived features, it is possible to observe a distinction between the behavior of indicators from wheel flats on the right and left sides. It is relevant to mention that only one sensor is adequate to detect a defective wheel using the AR-derived features.

#### 4.4.2. ARX Model

**Figure 21:**
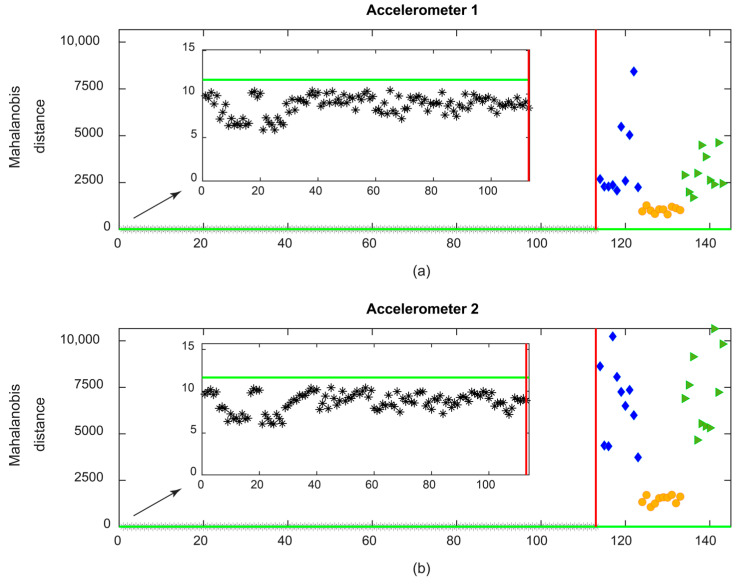

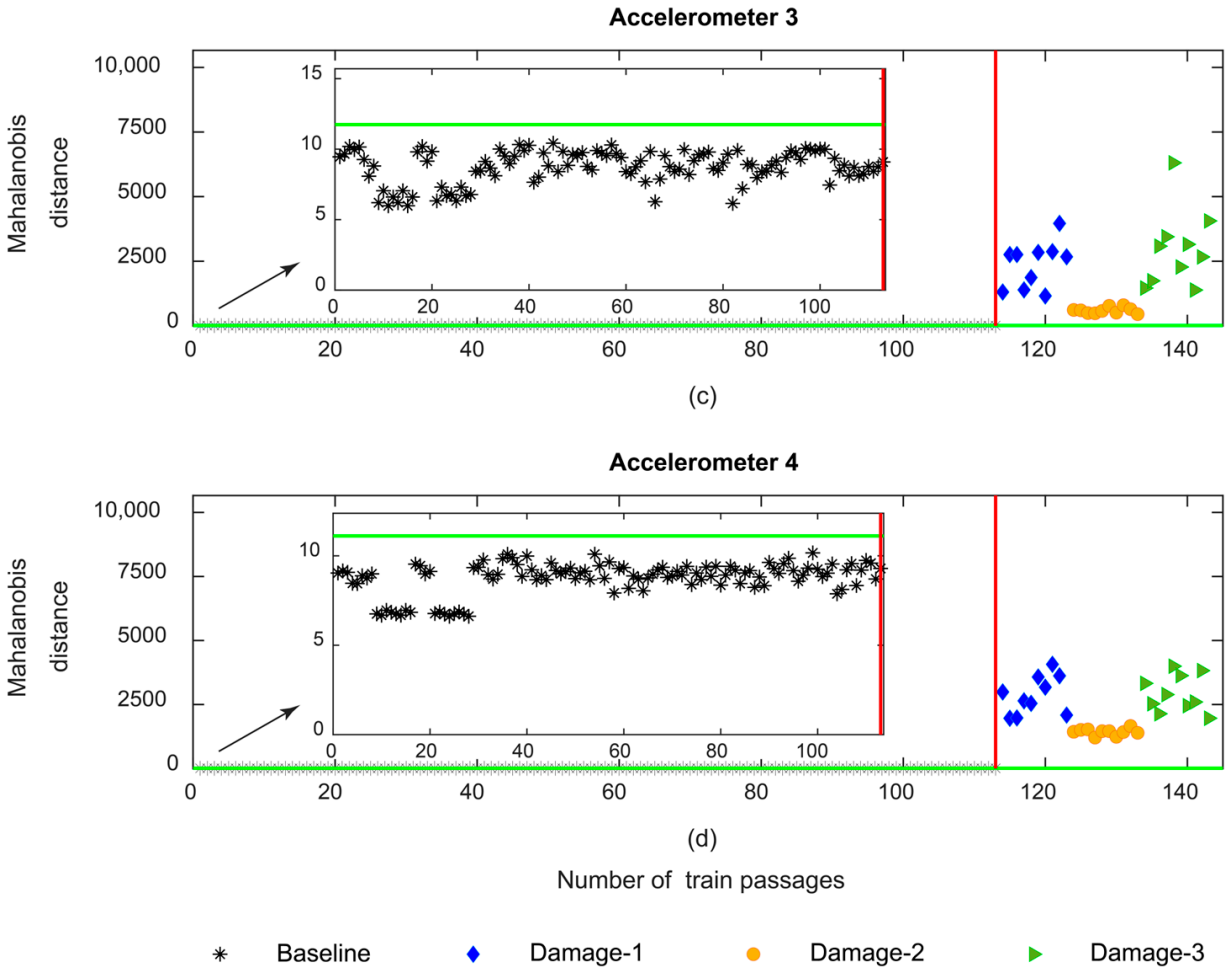
ARX—automatic wheel flat damage detection considering the responses from accelerometers 1–4: (**a**) MD for accelerometer 1, (**b**) MD for accelerometer 2, (**c**) MD for accelerometer 3, (**d**) MD for accelerometer 4.

The results of the automatic damage detection for the ARX-derived features are presented in Figure 21. It can be observed that the extracted features can effectively detect all the damage scenarios without the occurrence of any false positives or negatives. Additionally, from the accelerometers located on the sleeper it is possible to detect the damages (Figure 21d). This conclusion is particularly relevant since it is a clear advantage in relation to the performance of the AR model, and because installing sensors on the sleeper is easier than installing on the rail. Additionally, the ARX method is also promising in terms of its ability to distinguish between damaged wheels on the left or right sides. Another advantage of using the ARX method is that this technique can detect defective wheels without any false positives or negatives, regardless of the sensor’s position. Finally, in the case of ARX, it should be mentioned that installing one sensor is sufficient to distinguish a healthy wheel from a defective one.

#### 4.4.3. CWT

**Figure 22:**
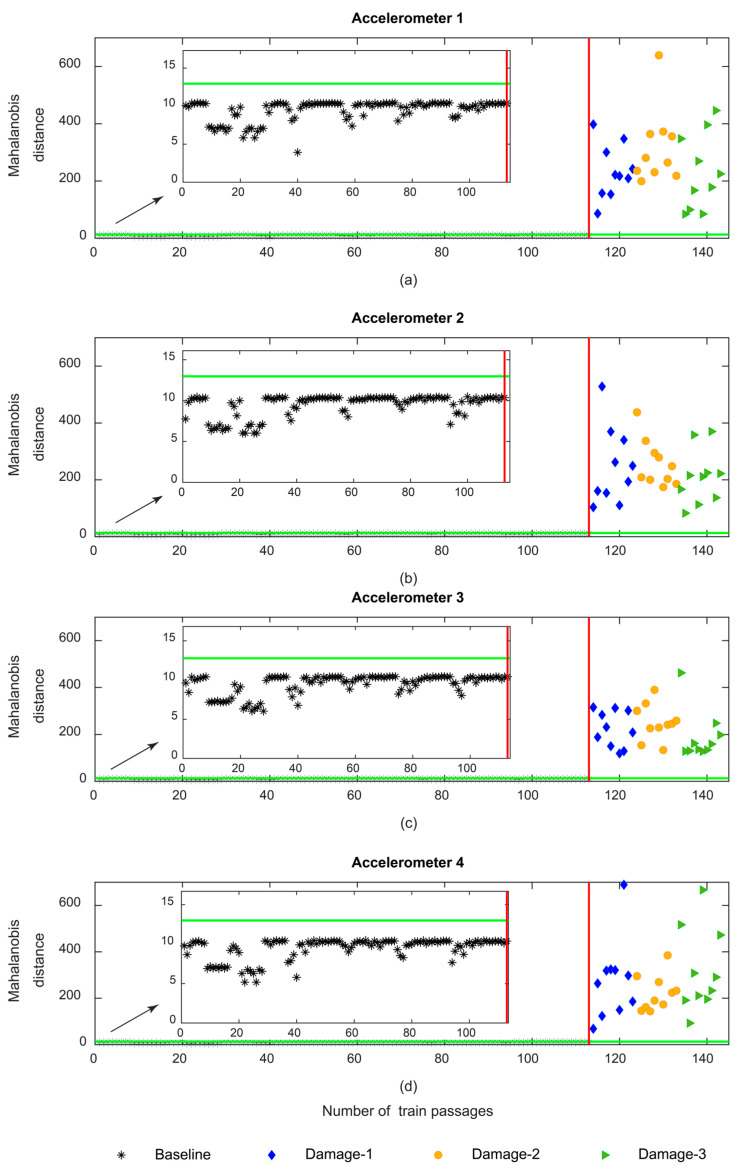
CWT—automatic wheel flat damage detection considering the responses from accelerometers 1–4: (**a**) MD for accelerometer 1, (**b**) MD for accelerometer 2, (**c**) MD for accelerometer 3, (**d**) MD for accelerometer 4.

Figure 22 illustrates the damage detection assessment based on CWT-derived features. It is possible to infer that, automatic damage detection can provide an accurate distinction between the baseline and damaged scenarios without any false positives or negatives. Moreover, by locating the accelerometers on the sleeper only, damage detection using CWT is possible, in addition to the simplicity of installation. However, it can be concluded that damage detection by implementing the CWT-derived features is not sensitive to the side of the defect. Nevertheless, as in previous features, only one sensor is necessary to distinguish a defective wheel from a healthy one.

#### 4.4.4. PCA

**Figure 23:**
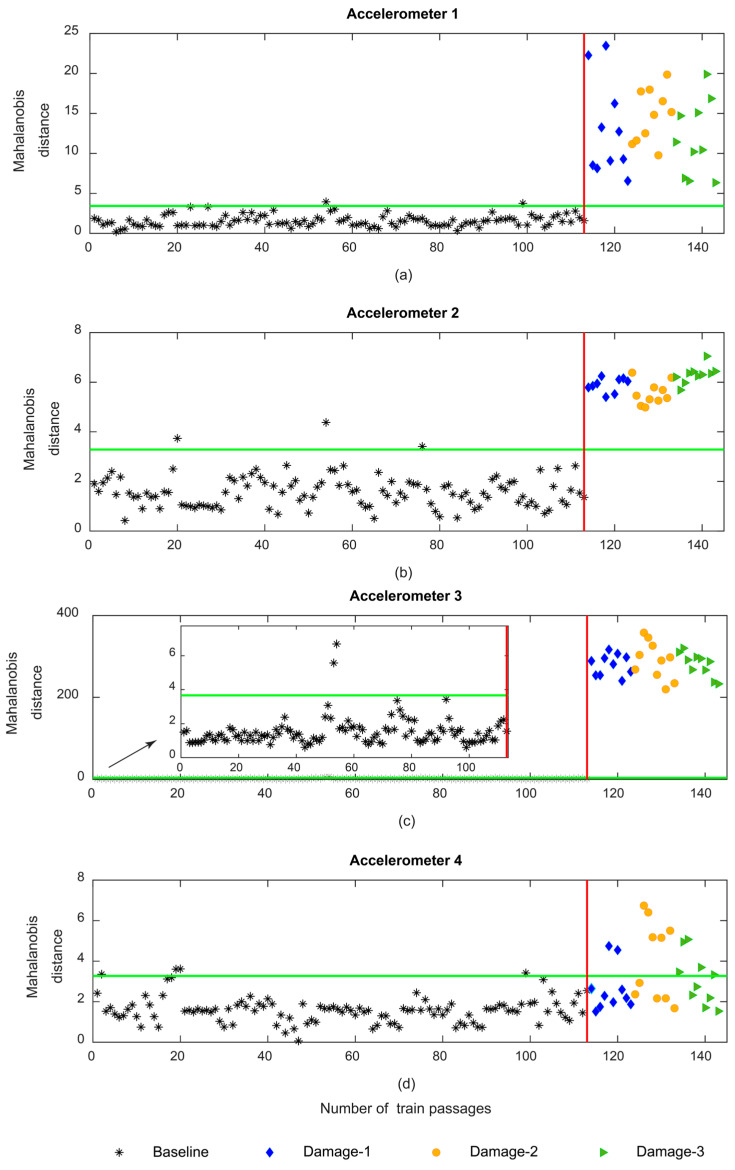
PCA—automatic wheel flat damage detection considering the responses from accelerometers 1–4: (**a**) MD for accelerometer 1, (**b**) MD for accelerometer 2, (**c**) MD for accelerometer 3, (**d**) MD for accelerometer 4.

Figure 23 represents the automatic damage detection based on PCA-derived features for sensors 1–4. As shown in this figure, damage detection comes with at least two false positives. The output of damage detection based on the PCA-derived features lacks robustness and is not able to properly detect damaged wheels. In comparison to the AR-, ARX- and CWT-derived features, the PCA has less accuracy in damage detection.

**Figure 24:**
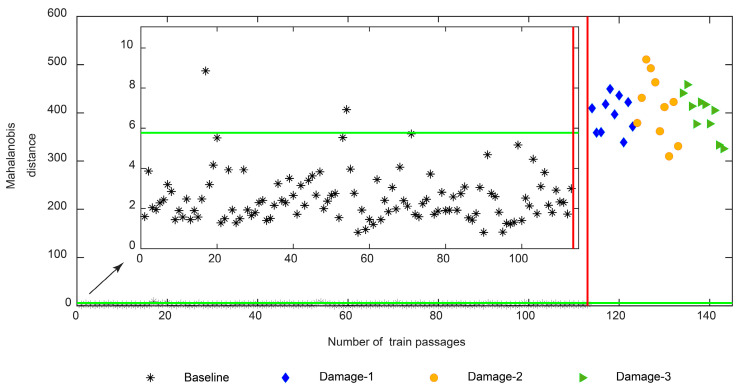
PCA—automatic wheel flat damage detection considering the multi-sensor data fusion.

From Figure 23, it can be concluded that the automatic damage detection based on PCA-derived features lacks robustness and the output comes with false positives and negatives. Therefore, to enhance the sensitivity to defects, the second stage of the data fusion consisting of multi-sensor fusion is implemented, by using data from sensors on both sides of the track. As shown in Figure 24, it is visible that after the second stage of data fusion the PCA-derived features come without any false negatives. On the other hand, the number of false positives is reduced to only two.

## 5. Conclusions

This study aimed to compare the accuracy of an unsupervised data-driven methodology, based on four distinct features (AR, ARX, CWT, and PCA), for the automatic detection of wheel flats and based on time–history accelerations on the track elements (rails and sleepers).

The proposed methodology includes (i) feature extraction from acquired responses using dedicated feature extraction methods; (ii) feature normalization based on principal component analyses (PCA); (iii) data fusion to merge features derived from each sensor and (iv) damage detection by performing an outlier analysis using a specific confidence boundary. 

From the research presented herein, it is possible to draw the following conclusions:

- the AR and ARX methods are the most accurate feature extraction methods for wheel flat damage detection as they can robustly detect defects; these two methods are sensitive to the side of the damage being the most promising to automatically distinguish an existing defective wheel on the right side from the left side in future works;
- the CWT method is only capable of detecting damaged wheels and is not sensitive to the side of the defect;
- the accuracy of the PCA method to detect the defective wheel is low and damage detection using this method lacks reliability;
- the ARX method is the only method that can robustly detect the wheel flat with accelerometers placed in the sleepers.
- One of the novelties of this research in relation to previous works [5,8] is the comparison of the accuracy of four different feature extraction techniques using an unsupervised learning methodology to automatically detect a defective wheel, which is a clear step forward in terms of the effectiveness of the proposed method, and allows full implementation for real-world applications.

Such results clearly show the great potential of this innovative application of data mining in the railway industry, particularly for infrastructure managers. Future work includes a field trial to validate the proposed methodology based on on-site measurements. Furthermore, for the final development of the proposed methodology, it is imperative to develop a novel feature, or eventually upgrade the actual methodology, to additionally classify the severities of the wheel flats.

## Figures and Tables

**Table 1:** Damaged and undamaged scenarios.

	Baseline Scenarios	Damaged Scenarios
Train	Freight—Laagrss wagon	
Number of loading schemes	6	1 (full capacity)
Unevenness profiles	4	1
Speeds (km/h)	40–120	80
Noise ratio	5%	
Flat lengths (mm)	−	50–100
Number of numerical analyses	100	30

## Data Availability

All data: models: and code generated or used during the study appear in the paper.
